# Detection of circulating tumor cells in non-metastatic prostate cancer through integration of a microfluidic CTC enrichment system and multiparametric flow cytometry

**DOI:** 10.1371/journal.pone.0312296

**Published:** 2024-10-23

**Authors:** Meltem Kilercik, Ebru Özgür, Şebnem Şahin, Begüm Şen Doğan, Ege Mutlu, Cenay Cihan, Murat Kolay, Nilüfer Erkal, Özge Zorlu, Tünkut Salim Doğanca, Ali Rıza Kural, İlter Tüfek, Haluk Külah

**Affiliations:** 1 Department of Biochemistry, Acibadem University, Istanbul, Turkiye; 2 Mikro Biyosistemler A.S., Ankara, Turkiye; 3 Acıbadem Labmed Clinical Laboratories, Istanbul, Turkiye; 4 Urology Clinic, Acibadem Taksim Hospital, Istanbul, Turkiye; 5 Department of Urology, Acibadem University, Istanbul, Turkiye; 6 Department of Electrical and Electronics Engineering, Middle East Technical University, Ankara, Turkiye; West China Hospital, Sichuan University, CHINA

## Abstract

Prostate cancer (PCa) is the second most common cancer among men and the fifth leading cause of cancer death. Circulating tumor cell (CTC) enumeration and characterisation in PCa has been shown to provide valuable information on prognosis of disease, therapy management and detection of resistance. Here, Cellsway’s microfluidic platform for high-throughput enrichment of intact CTC populations was used to isolate CTCs from the blood of 20 localised PCa patients and 10 healthy donor samples to evaluate the clinical performance of the technology. To enumerate and characterise CTCs, a multi-parameter flow cytometry analysis was performed on the enriched CTC suspension using CTC-specific biomarkers. CTCs were detected in 17 of 20 patient samples, which corresponds to 85% CTC positivity. The median CTC count per 7.5 ml blood was 2 (1–9). In 80% of patients (n = 16), the number of CTCs ranged from 1 to 5, and in 5% of patients (n = 1) the number of CTCs was above 5. No CTCs were observed in the blood samples of 10 healthy volunteers, demonstrating the high specificity and low risk of false positives of the technology.

## Introduction

Globally, prostate cancer (PCa) is the second most frequent cancer and the fifth leading cause of cancer death among men with an estimated >1.4 million new cases and >375.000 deaths in 2020 [1]. The prostate-specific antigen (PSA) and digital rectal exam (DRE) are tests for the initial clinical assessment of the prostate for the presence of cancer. An abnormal DRE or an elevated PSA test may indicate that a tissue biopsy is needed. The current gold standard diagnosis of PCa is based on tissue biopsy, which is the removal of tissue from the prostate to assign the Gleason score and a Gleason grade group. The risk group, prognosis, and treatment are then based on clinical and pathological parameters including, tumor stage, Gleason score, and PSA concentration [2, 3].

PSA is a protease secreted by the epithelial cells of the prostate gland and its secretion is often increased during PCa. Although PSA has been the most commonly used biomarker for PCa management for decades, it has an important limitation: low specificity. PSA levels can also be elevated in many non-malignant diseases of the prostate, such as prostatitis and benign prostatic hyperplasia (BPH) [4]. PCa is a heterogeneous disease, which is not limited only to differences between patients. In a single patient, the primary tumor and the metastatic lesions or even different sites of the same tumor can demonstrate variations in molecular profiles. Highly invasive tissue biopsy gives only a snapshot of the tumor at a certain location and time [5, 6]. Therefore, a method is needed to enable reliable and real-time monitoring of changes occurring in the tumor and treatment response. Circulating tumor cells (CTCs) are cancer cells that detach from the primary or metastatic tumors entering blood through intravasation. Isolation of CTCs via liquid biopsy offers a readily accessible source of cancer cells representative of the heterogenous tumor material and overcomes the limitations of tissue biopsy.

The enumeration and characterization of CTCs in metastatic PCa have been demonstrated to provide valuable information about disease prognosis, therapy management and detection of resistance [7–10]. CTC enumeration at baseline and during treatment was shown to be a strong independent prognostic marker to predict disease progression and overall survival in metastatic PCa [11–13] and was found to be more prognostic than PSA [14, 15]. Characterization and molecular analysis of CTCs provide additional information in the realization of personalized therapy in metastatic PCa [16–18].

CTCs are known to be the main player in metastasis, and they represent a promising prognostic biomarker for better prediction of risk for recurrence in non-metastatic PCa compared to standard prognostic biomarkers [19]. However, the number of studies on CTCs with non-metastatic (localized and locally advanced) PCa are limited because of technical challenges in CTC isolation due to the rarity (1 CTC/1 × 10^9^ blood cells) and heterogeneity of CTCs [15, 20, 21].

Microfluidic systems have a lot of promise for high-efficiency CTC isolation. The advantages and limitations of different methodologies for CTC isolation that utilize microfluidics have been detailed elsewhere [22–24]. Due to the highly heterogeneous nature of CTCs, isolation technologies based on biological differentiating properties like surface biomarkers are not reliable and usually yield low CTC numbers. On the other hand, epitope-independent isolation techniques that utilize physical differences between blood cells and CTCs provide a heterogeneous CTC suspension reflecting the clonal heterogeneity within a primary tumor or between different metastatic sites.

Cellsway’s microfluidic CTC enrichment platform utilized in this study provides high recovery, intact CTC isolation from a blood sample, without a loss of cell viability and with a short sample processing time [25]. The enrichment process is based on size differences of cancer cells from other blood cells and does not rely on the expression of certain cell surface molecules enabling the capture of heterogeneous CTC populations. The technology has been validated via analytical studies using spiked blood samples which show 77% recovery at a single-step process when Michigan Cancer Foundation-7 (MCF7) breast cancer cell lines are utilized. This study has been designed to evaluate the clinical performance of the technology in isolating CTCs from blood samples of PCa patients.

In this study, Cellsway’s microfluidic platform that delivers high-efficiency enrichment of intact CTC populations was used to isolate CTCs from the blood of 20 localized PCa patients, together with 10 healthy donor samples, to evaluate the clinical performance of the technology. A multiparameter flow cytometry analysis using CTC specific biomarkers was carried out on the enriched CTC suspension to enumerate and characterize CTCs. Multiparameter flow cytometry is a powerful analytical tool that enables high-throughput and simultaneous measurement of multiple phenotypical characteristics of cells [26]. It allows detection of rare cell population, including CTCs, in blood together with their multiphenotypical properties. It can be incorporated or adapted to any tumor type. Thanks to advanced analytic strategies, it can now be used to isolate and characterize tumor cells in circulation as low as 0.0001% [27].

## Materials and methods

### Clinical samples

Twenty non-metastatic PCa patients and age-matched 10 healthy volunteers as control were included in the study. Ethical approval for blood collection was taken from the Ethical Committee of Acıbadem University Hospital, İstanbul, Turkey, and the studies performed were in accordance with the ethical regulations under ethical committee-approved protocols (Protocol code #MBS-CTC-HEU-01). Samples were collected between 29.04 2021 and 11.02 2022. A written informed consent form was obtained from each donor before participating in the study. Peripheral blood samples (7.5 ml) were collected in a K_2_EDTA blood collection tubes (BD Vacutainer, Cat# 367525, USA) after discarding the first 2.0 ml of blood, in order to avoid potential contamination with skin epithelial cells. Samples were stored at room temperature and processed within 4 hours of the collection. All blood samples were taken 1 hour prior to operation. All patients had undergone robot-assisted radical prostatectomy and bilateral pelvic lymph node dissection. None of the patients received neoadjuvant chemotherapy. Final histopathology results of the patients are given in Table 1. Collected samples were processed via the microfluidic CTC enrichment system and analyzed via flow cytometry for CTC counting and characterization.

**Table 1:** Final histopathology results of the patients.

Prostate Ca pts (n:20)	Age (Med)	Tumor V (Mean ± SD)	Gleason Score-(n)	Grade group (n)	Final histopathology (n)
	63 (47–75)	5.36 ± 5.4	7 (3+4)-(14)	2 (14)	
			7 (4+3)-(3)	3 (3)	pT2 pN0 (13)
			9 (4+5)-(2)	5 (1)	pT3a pN0 (6)
			6 (3+3)-(1)	1 (1)	pT3a pN1 (1)

### Blood processing

The whole blood (7.5 ml) was processed with the density gradient centrifugation method (Ficoll Paque Plus, GE Healthcare, USA) to get rid of red blood cells (RBCs) and collect peripheral blood mononuclear cells (PBMCs) and CTCs from the buffy coat. Briefly, anticoagulant (EDTA) treated blood was diluted with an equal volume of phosphate-buffered saline (PBS) and mixed by pipetting. Diluted whole blood was carefully layered on top of Ficoll Paque Plus and centrifuged according to the manufacturer’s instructions. The buffy coat containing PBMCs and CTCs was collected and transferred to a clean centrifuge tube. After a single wash with PBS, the supernatant was discarded and the cells were resuspended in 10 ml of PBS. Before the microfluidic CTC enrichment process, the cell suspension was passed through a 30 μm cell strainer (Miltenyi Biotec, Germany) to remove the impurities and prevent clogging.

### Microfluidic CTC enrichment system

**Fig 1:**
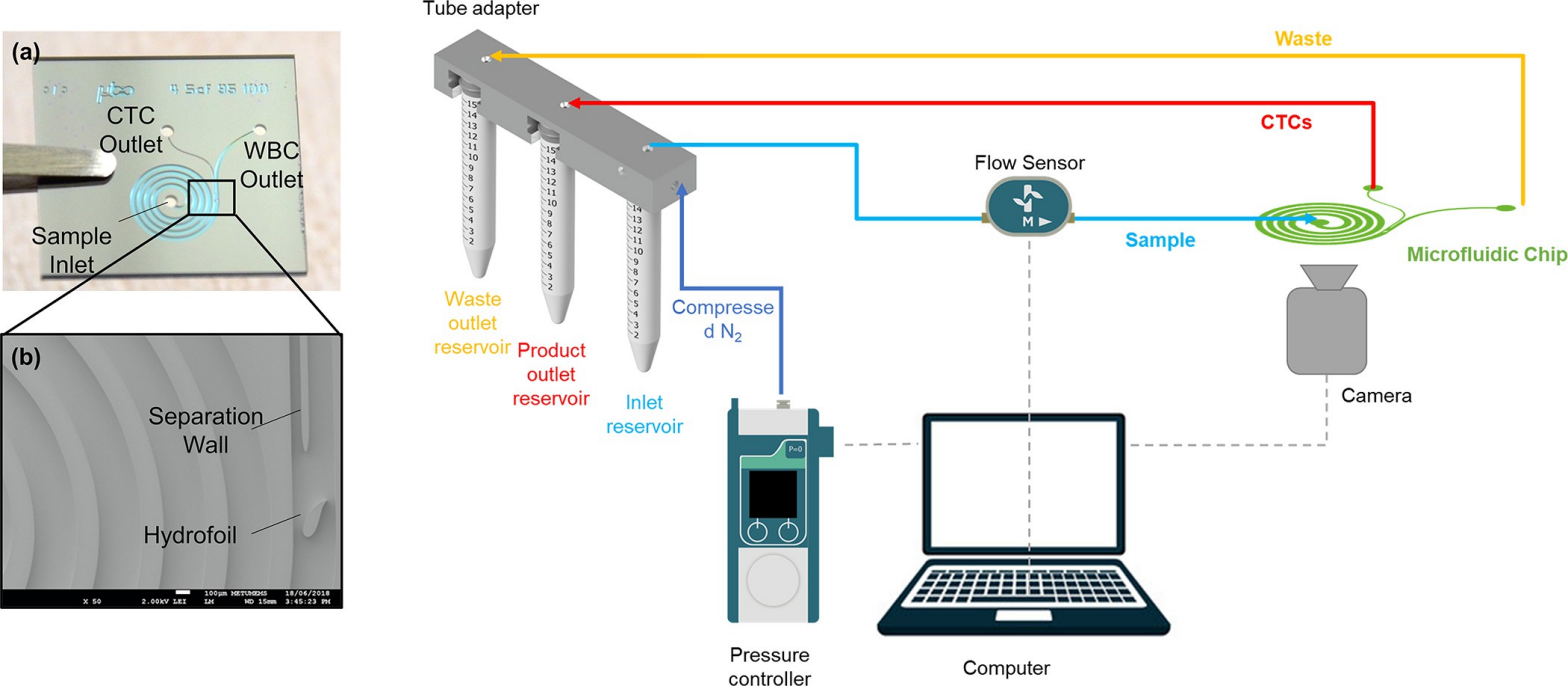
The microfluidic chip and the operational setup.

The chip utilizes a spiral microfluidic channel structure for hydrodynamic, size-based separation of CTCs from blood cells. The channel is integrated with a hydrofoil structure at downstream of the spiral that enhances the separation efficiency by increasing the distance between streams of CTCs and blood cells [25]. The channel is fabricated through a MEMS process utilizing silicon and glass substrates. The microfluidic chip is mounted in a custom-designed chip holder that facilitates microfluidic connections for fluid flow. A pressure-driven flow was provided to the channel using a pressure controller (Fluigent, France). The microfluidic chip and a schematic of the operation setup is presented in Fig 1.

### Sample processing

Before processing the blood samples for CTC enrichment, the chip surface was conditioned using ethanol and DI water to prevent air bubbles. Then, the microchannel was filled with an anti-biofouling agent, PLL-g-PEG [poly(L-lysine-[g]-poly(ethylene glycol), (SuSoS AG, Switzerland], and incubated for 30 mins to prevent cell adhesion. After washing with PBS, the sample was fed into the channel at a flow rate of 1.2 ml/min. The product outlet was collected and analyzed by flow cytometry after measurement of the total cell concentration to estimate the white blood cells (WBCs) depletion ratio. To calculate the WBC depletion rate of the system, the number WBCs in the inlet sample and the WBCs in the product reservoir were compared. WBC concentration of the inlet (before the process) and product samples were calculated via a hemogram device. Since the numbers of WBCs are extremely higher than the number of CTCs, it is assumed that the cell count gives the WBC concentration of the sample. The depletion rates were calculated by the equation given below.


$$WBC\;Depletion\;Rate\;(\%)=\left(\frac{WBC\;\#\;in\;waste}{WBC\;\#\;in\;product+WBC\#\;in\;waste}\right)x\;100$$

### Phenotypic characterization of CTC by flow cytometric method

The collected samples were analyzed by flow cytometry after immunofluorescent (IF) labelling to identify PCa CTCs and eliminate peripheral cells. We used a combination of markers including pan-cytokeratin, epithelial cell adhesion molecule (EpCAM), cytokeratins, and stem cell biomarkers (CD44, CD117) to identify CTCs from epithelial origin in the blood. This combination was complemented with 7-AAD (BD Biosciences, CA, 559925) to select nucleated cells, and CD45 to exclude white blood cells [28]. The following antibodies were used for IF labelling: Anti-CD45-PerCP-Cy5.5 (BD Biosciences, 332784, clone 2D1), Anti-EpCAM-PE (BD Biosciences, 347198, clone EBA-1), Anti-CD44-FITC (BD Biosciences, 560977, clone G44-26), and Anti-CD117-PE/Cyanine7 (Biolegend, CA, 313212) antibodies, 7AAD (BD Biosciences, 559925). The cell suspension was transferred into a FACS flow tube and incubated with the antibody cocktail at room temperature for 20 min. After incubation, FACS perm/fix solution applied and Anti-pan-Cytokeratin-AF647 (BD Biosciences, 563648, clone KA4) was added and incubated for 15 min. To get rid of the remaining erythrocytes, FacsLyse (2 ml, BD Biosciences, 349202) solution was added into the suspension, vortexed and incubated at room temperature for 10 min. Tubes were centrifuged at 540 x g for 5 min, the supernatant was removed and Cellwash solution (2 ml, BD Biosciences, 349524) was added to the pellet. The cell suspension was washed at 540 x g for 5 min and Facsflow (350 μl, BD Biosciences, 342003) was added to the cell pellet and the cell suspension was analyzed with FACSCanto II flow cytometer (BD Biosciences) utilizing Diva software [29]. All the events were acquired on low flow rate.

Potential CTCs were initially identified as 7-AAD+, pan-cytokeratin+, CD45-, EPCAM+/- cells. By the use of these fluorescent markers and a gating strategy analysis of events, we were able to identify our target cells for the best possible rare event detection.

### Statistical analysis

In our study, Spearman’s rank correlation coefficient was computed to assess the relationship between CTC count and serum PSA level, tumor volume, tumor %, Gleason score, and grade group of 20 patients (Table 1) using SPSS (24.0 for Windows) program (CI:95%, p<0.05).

## Results

Blood samples of non-metastatic PCa patients and healthy controls were processed through the microfluidic CTC enrichment system. The system is extremely high throughput, with a processing time of only 10 minutes for 7.5 ml of blood per individual. The product of the enrichment system resulted in a WBC depletion ratio of 68% ± 13%, at a single-step process (S1 and S2 Tables in S1 File), which is suitable for flow cytometric downstream analysis. For downstream analyses requiring a higher purity, multistep processing is possible by reprocessing the sample with the microfluidic system [25].

Patient information, clinical parameters and CTC counts are shown in Table 2. CTCs were detected in 17 of 20 patient samples corresponding to 85% CTC positivity. The detected number of CTCs varied between 1 and 9, with a median value of 2. The number of CTCs in 80% of the patients (n = 16) varied between 1–5, and 5% of the patients (n = 1) had a CTC count above 5 per 7.5 ml of blood sample (Table 3). We did not observe any CTCs in blood samples of 10 healthy volunteers demonstrating the technology’s high specificity and low risk of false positives.

**Table 2:** CTC enriched with tagCTC-ENR process and detected with FACS flow cytometry analysis from localized PCa patients.

Patient No	Stage	Age	PSA (ng/ml)	Tumor V (cc)	Tumor %	Gleason Score	Grade Group	CTC Count
**1**	pT2 pN0	69	1.75	0.1	0.12	6 (3+3)	1	**0**
**2**	pT2 N0	63	2.8	2	5	7 (3+4)	2	**3**
**3**	pT2 pN0	59	5.9	2	5	7 (3+4)	2	**1**
**4**	pT2 pN0	52	3.3	0.9	1.5	7 (3+4)	2	**3**
**5**	pT2 pN0	58	6.98	1	2	7 (3+4)	2	**2**
**6**	pT2 pN0	47	1.9	1.75	5	7 (3+4)	2	**2**
**7**	pT2 pN0	61	8.7	5	11	7 (3+4)	2	**0**
**8**	pT2 pN0	75	12.6	4.62	11	7 (3+4)	3	**3**
**9**	pT2 pN0	56	3.6	1.64	4	7 (3+4)	2	**2**
**10**	pT2 pN0	58	9.8	8	28	7 (3+4)	2	**3**
**11**	pT3a pN0	55	9.1	6.24	12	7 (3+4)	2	**1**
**12**	pT3a pN0	69	13	5	21	7(3+4)	2	**1**
**13**	pT3a pN0	74	5.88	9.24	22	7 (3+4)	2	**3**
**14**	pT3a pN0	63	4	5	10	7 (3+4)	2	**3**
**15**	pT3a pN0	59	6.2	4	13	7 (3+4)	2	**1**
**16**	pT2 pN0	57	3.74	4.2	12	7 (4+3)	3	**0**
**17**	pT2 pN0	73	11.5	4.6	5	7 (4+3)	3	**2**
**18**	pT2 pN0	69	7.4	2.8	8	7 (4+3)	3	**2**
**19**	pT3a pN0	65	5.2	19.2	40	9 (4+5)	5	**9**
**20**	pT3b pN1	63	20	20	37	9 (4+5)	5	**1**

**Table 3:** The range of CTCs detected in non-metastatic PCa patients. A 85% CTC positivity were detected in total of 20 patient samples. 16 patients (80%) had a CTC count below 5, 1 patient (5%) had CTC counts above 5, and 3 patients had no CTCs. No CTCs were detected in control samples.

	No CTC	1–5 CTCs	>5 CTCs
PCa (n = 20)	3 (15%)	16 (80%)	1 (5%)
Healthy male (n = 10)	10 (100%)	-	-

## Discussion

It is known that CTCs have a great potential in the management of PCa [19]. However, the number of studies conducted on CTCs in patients with localized PCa is less than those with metastatic PCa mainly due to the technical limitations in isolation of a low number of CTCs especially at the early stage [17, 19]. In studies carried out on non-metastatic PCa, CTCs were only detected in a limited number of patients [15, 30].

Radical prostatectomy, removal of the entire prostate, is an important treatment option for localized PCa patients. Unfortunately, about 30% of patients develop recurrence after prostatectomy [31]. A recent study showed that the prediction of recurrence by detection of CTCs could enable better patients’ stratification for surgical treatment and adjuvant treatment after prostatectomy compared to PSA [32]. Besides enumeration, the characterization of CTCs by downstream analysis provides additional prognostic and predictive information in localized PCa [33]. Salami et al. 2010 demonstrated that the detection and characterization of CTCs may provide useful information for risk stratification and the need for multimodal therapy in newly diagnosed localized high-risk PCa patients [33]. Liu et al. suggested that the presence of the CTCs was highly correlated with the clinical parameters and they could aid in the prediction of the pathology outcomes before prostatectomy [34]. In addition to that, Castillo et al. reported that more than 3 CTCs per 6 mL of a blood sample, was significantly associated with the risk of recurrence [32].

In this study, flow cytometry was chosen as a downstream analysis technique of CTCs since it is known that, when combined with a good enrichment method, multiparametric flow cytometric analysis could significantly improve the characterization of CTCs in breast and PCa patients [35]. The enrichment technology utilized in this study does not rely on the presence of cell surface antigens, thus enabling high recovery enrichment of heterogeneous CTCs subpopulations [25]. These properties make the system compatible with different downstream analyses for the detection of a variety of biomarkers that have clinical utility for PCa management.

In this study, cytokeratins (pan-CK) were used as inclusion markers to identify CTCs, while CD45 was used as an exclusion marker to exclude non-tumor cells, particularly leukocytes. EpCAM, CD44, and CD117 biomarkers were used for further characterization of CTCs. EpCAM is a type I glycosylated membrane protein expressed at low levels in a variety of human epithelial tissues, but overexpressed in most solid cancers, including prostate cancer [36] and most of the studies reported to date use EpCAM as the target marker to identify potential CTCs. Its expression has been demonstrated to be inversely related to cancer patients’ prognosis [37]. However, EPCAM is specific for well-differentiated epithelial cells and is one of the first epithelial markers lost on CTCs during the epithelial-to-mesenchymal transition (EMT) process before other epithelial markers which is associated with increased invasiveness and metastasis of cancer cells [28].

To identify CTCs that has undergone EMT, CD44 and CD117 were added to CTC analysis. CD44, a single-pass type I transmembrane glycoprotein located on the cell surface. It is involved in cell adhesion, facilitates communication and interaction between cells, plays a significant role in promoting tumor cell invasion, migration and metastasis, and also may enhance cell migration during EMT [38, 39]. Studies have shown that increased CD44 expression is associated with advanced prostate cancer stages, higher Gleason scores and poorer prognosis. Inclusion of CD44 as a biomarker enables identification of CTCs that possess a more complete EMT molecular profile. CD177, a member of the type III tyrosine kinase receptor family, is involved in cell signaling and maintains a variety of cell functions, including survival, metabolism, growth and progression, proliferation, apoptosis, migration, and differentiation. Some studies suggest that CD117 expression may be associated with aggressive prostate cancer phenotypes and poorer prognosis [39, 40]. It is a potential marker used for identification of cancer stem-like cell subpopulations. Consequently, CD117 immunophenotyping was included to provide further details on the molecular characteristics of CTCs.

Table 4 summarizes CTC studies conducted using localized PCa patient samples utilizing different CTC enrichment and identification technologies. These studies shows that CTC positivity rate of localized PCa patients varies between 11–75%, which indicates a wide range of variation depending on the technology used. The rate of CTC positivity in this study was 85% (17 out of 20), which is higher than the other studies utilizing epitope-independent CTC enrichment technologies conducted in non-metastatic PCa. Size-based enrichment methods demonstrate similar rates of CTC positivity in the patient with localized PCa, ranging between 50–55% [32, 34, 41, 42]. Other methods such as immunoaffinity, flow-cytometry or negative selection-based technologies have a broader scale between 11% to 75% [10, 30, 43, 44].

**Table 4:** A summary of CTC enrichment and isolation studies conducted using localized PCa patient samples.

CTC Isolation Technology	Approach	Clinical Stage	Number of Patients	Number of CTC+ Patients (%)	Median (Range) CTC/ml	Reference
Epic Sciences	No enrichment	T2c or greater	45	33 (73.3%)	1.3 (0⎼22.5)	[33]
A microfluidic ratchet chip	Size-based	37 T2, 13 T3	50	25 (50%)	4.5 (0.5⎼208.5)	[41]
MetaCells filtration chip	Size-based	45 T2 10 T3	55	28 (52%)	NA	[42]
CanPatrol CTC enrichment	Size-based	5T1, 71 T2,4 T3	80	44 (55%)	NA (0.2⎼2.6)	[34]
ISET	Size-based	65 T2, 43 T3	108	55 (50.9%)	NA, (0.16⎼2.3)	[32]
Cellsearch	Immunoaffinity (EpCAM-based)	95 T2, 57 T3	152	17 (11%)	0.13 (0.13⎼13.3)	[30]
CellSearch	Immunoaffinity (EpCAM-based)	37 T1, 45 T2, 4 T3	86	33 (38.4%)	0.24 (0.13⎼1.3)	[43]
CellCollector	EpCAM based			52 (62.7%)	0.32 (0.13⎼1.6)	
EPISPOT	Negative selection-based			45 (52.9%)	0.4 (0.13⎼1.7)	
CTC-Chip	Immunoaffinity (EpCAM based)	NA	19	8 (42%)	95 (38⎼222)	[44]
Fluorescence Activated Cell Sorting (FACS)	Flow cytometry-based	26 T2, 13 T3, 1 Tx	40	30 (75%)	10 (1⎼439)	[10]
**Cellsway Microfluidic CTC enrichment**	Size-based enrichment integrated with flow cytometry	13 pT2 N0, 6 pT3 N0, 1 pT3 N1	20	17 (85%)	0.27 (0–1.2)	This Study

In this study, Spearman’s rank correlation analysis was conducted to explore the relationship between CTC count, tumor volume, tumor %, Gleason score and grade group in prostate cancer patients. The analysis revealed a moderate positivity between CTC count and Gleason score (r = 0.31). However, this correlation is not statistically significant (p-value = 0.18). This suggests although there may be a weak trend of higher Gleason scores being associated with increased CTC counts, the relationship is not strong enough to be concluded from this dataset. The analysis did not reveal any significant correlation between the number of CTCs and serum PSA levels (r = -0.11, p = 0.63), tumor volume (r = 0.16, p = 0.49), tumor % (r = 0.09, p = 0.70), or grade group (r = 0.10, p = 0.49) (S3 Table in S1 File). In a study (Shao et al.), a significant association between CTC counts and T stage was found, their results showed that pT3 patients have higher CTC numbers than pT2 cases. Also, Castillo et al. reported that, patients with T2b tumors or higher stage had a significantly higher rate of CTC positivity than patients with T2a tumors [32]. However, they did not find a significant difference between patients with T2b, T3a and T3b tumors in CTC positivity. Nevertheless, Todenhöfer et al. did not find any correlation of CTC number with tumor stage or CTC presence with PSA concentration, age, tumor stage, lymph node stage, Gleason score, and risk category [41]. Similarly, Meyer and colleagues found no significant correlation between CTC presence and T-stage, Gleason score or serum PSA level [30]. Additionally, they reported that CTC positivity and biochemical recurrence did not correlate after 48 months of median follow-up. The reason for not finding a correlation between CTC count and the variables mentioned could be attributed to the small sample size of the study, which might not have been sufficient for conducting a proper statistical analysis. Larger studies may be needed to better understand the relationship between CTC count and tumor characteristics in prostate cancer. However, the lack of correlation does not diminish the clinical significance of the study. This is because the true clinical benefit will be derived from the molecular characterization of CTCs. The information obtained by molecular characterization may enable clinicians to perform targeted and personalized treatments, resulting in improved treatment outcome.

It has been shown that the microfluidic CTC enrichment system can be readily integrated with a flow cytometric counting and profiling of CTCs. As a future goal, studies should be conducted with a larger sample size to compare CTC profiles in different risk groups and include a follow-up period to test the potential of the technology for predicting the long-term outcomes and treatment responses of non-metastatic PCa patients.

## Supporting information

S1 File (DOCX)
