# Supplementary material for: Detection of circulating tumor cells in non-metastatic prostate cancer through integration of a microfluidic CTC enrichment system and multiparametric flow cytometry
Source: PLoS One. 2024 Oct 23;19(10):e0312296. doi: 10.1371/journal.pone.0312296 (PMC11498670; doi:10.1371/journal.pone.0312296)

**S1 Table.** **Total PBMC counts (as measured by the hemogram device) in patient samples and calculated WBC depletion rates.**

White Blood Cells (WBCs) depletion rate in clinical samples, after single step CTC enrichment process by Cellsway microfluidic system.

$$WBC Depletion Rate \left( \% \right)=\left( \frac{WBC \# in waste}{WBC \# in product+WBC\# in waste} \right)x 100$$

|  | **Total PBMC Counts** | | |  |
| --- | --- | --- | --- | --- |
| **Patient No** | **Inlet** | **Product Outlet** | **Waste Outlet** | **Depletion Rate (%) (W/(W+P))*100** |
| 1 | 6.64E+06 | 7.19E+05 | 2.76E+06 | 79.3% |
| 2 | 7.42E+06 | 4.55E+06 | 2.46E+06 | 35.1% |
| 3 | 7.43E+06 | 3.97E+06 | 9.06E+06 | 69.5% |
| 4 | 5.79E+06 | 1.02E+06 | 4.86E+06 | 82.7% |
| 5 | 1.35E+07 | 4.94E+06 | 7.18E+06 | 59.2% |
| 6 | 8.89E+06 | 5.44E+06 | 6.26E+06 | 53.5% |
| 7 | 2.57E+06 | 9.58E+05 | 2.68E+06 | 73.7% |
| 8 | 5.98E+06 | 7.04E+05 | 3.77E+06 | 84.3% |
| 9 | 3.27E+06 | 9.22E+05 | 2.22E+06 | 70.6% |
| 10 | 8.57E+06 | 3.41E+06 | 7.08E+06 | 67.5% |
| 11 | 2.88E+06 | 8.00E+05 | 1.49E+06 | 65.0% |
| 12 | 9.23E+06 | 1.59E+06 | 4.46E+06 | 73.7% |
| 13 | 1.59E+07 | 6.20E+06 | 1.11E+07 | 64.2% |
| 14 | 2.95E+06 | 4.13E+05 | 1.46E+06 | 77.9% |
| 15 | 4.36E+06 | 2.33E+06 | 2.00E+06 | 46.2% |
| 16 | 1.12E+07 | 1.94E+06 | 9.45E+06 | 83.0% |
| 17 | 6.90E+06 | 1.53E+06 | 6.40E+06 | 80.8% |
| 18 | 5.92E+06 | 4.54E+06 | 9.06E+06 | 66.6% |
| 19 | 6.82E+06 | 3.42E+06 | 4.57E+06 | 57.2% |
| 20 | 5.88E+06 | 1.24E+06 | 3.48E+06 | 73.8% |
| **Average** | | | | **68.19%** |
| **Std Dev** | | | | **12.95%** |
| **Median** | | | | **70.1%** |
| **Range** | | | | **35.1 - 83.0%** |

**S2 Table. Cell suspension volumes at inlet, product outlet and waste outlets. Volumetric ratio of waste outlet to product outlet is 1.6, by design. Lower or higher values.**

|  | **Volumes (ml)** | | |
| --- | --- | --- | --- |
| **Patient No** | **Inlet (PBMC/CTC suspension** | **Product Outlet** | **Waste Outlet** |
| 1 | 9 | 3.3 | 5.2 |
| 2 | 9 | 3.5 | 5.25 |
| 3 | 9 | 3.5 | 5.5 |
| 4 | 9 | 3.7 | 5.5 |
| 5 | 9 | 3.5 | 5.5 |
| 6 | 9 | 3.6 | 5.5 |
| 7 | 9 | 3.1 | 5.5 |
| 8 | 8.8 | 3.2 | 5.4 |
| 9 | 9 | 3.6 | 5.5 |
| 10 | 9 | 3.5 | 5.5 |
| 11 | 9 | 4 | 5 |
| 12 | 9 | 3.5 | 5.5 |
| 13 | 9 | 3.5 | 5.5 |
| 14 | 9 | 3.5 | 5.6 |
| 15 | 9 | 3.75 | 5.5 |
| 16 | 9.3 | 3.7 | 5.8 |
| 17 | 9 | 3.5 | 5.5 |
| 18 | 9 | 4 | 5.5 |
| 19 | 9 | 3.5 | 5.1 |
| 20 | 9 | 3.6 | 5.1 |

**S3 Table. Spearman’s rank correlation coefficients between CTC count and PSA level, tumor volume, tumor %, Gleason score and grade group. CI:95%, p<0.05 was considered statistically significant.**

|  | **Spearman’s rank correlation cofficient (r)** | **p-value** |
| --- | --- | --- |
| PSA | -0.11 | 0.63 |
| Tumor Volume | 0.16 | 0.49 |
| Tumor % | 0.09 | 0.70 |
| Gleason score | 0.31 | 0.18 |
| Grade group | 0.19 | 0.42 |

**S1 Fig. – Methodology for the analysis of Flow Cytometry Data.**

A sample analysis from a patient sample to exemplify the methodology for the flow cytometry analysis.

Plot 1 (Ungated) - Gate drawn on Forward scatter vs. Side scatter plot to exclude debris.

Plot 2 (B) - Gate drawn on Forward scatter vs. 7-AAD to include only viable events

Plot 3 (Ungated) - Gate drawn on Forward scatter vs. CD45 to exclude bright CD45 positive cells

Plot 4 (C) - CD45 negative, EpCAM positive cells

Plot 5 (C) - CD45 negative, cytokeratin positive cells.

Plot 6 (C) - CD45 negative, EpCAM positive, cytokeratin positive cells ( < % 0.001)

Plot 7 (C) - CD45 negative, CD117 positive cells

Plot 8 (C) - CD45 negative, CD44 positive cells

Plot 9 (C) - CD45 negative, EpCAM positive, CD44 positive cells (<%0.001)


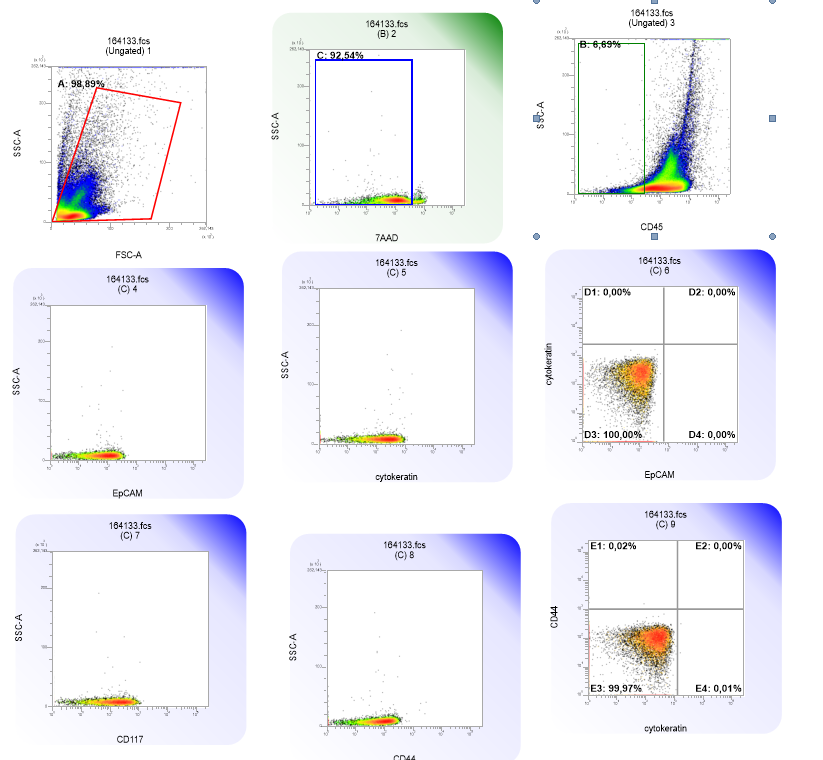


**S2 Fig. Scatter plots for the Isotype control, MCF7 breast cancer cell line and PBMC control of CD45 and EpCAM stained cell.**
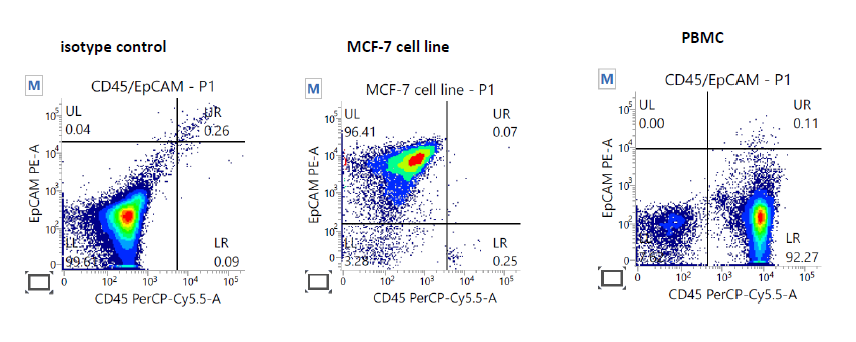

Supplement: S1 File — (DOCX) [file pone.0312296.s001.docx]
